# Role of Field Epidemiology in Environmental and Climate Change-Related Health Incidents in Wales: A Qualitative Analysis Through Expert Interviews

**DOI:** 10.3390/ijerph22091452

**Published:** 2025-09-18

**Authors:** Omer Faruk Sonmez, Behrooz Behbod, Christopher Roberts, Marco Barracchia, Astghik Baghinyan, Lichita Indra, Katarzyna Czabanowska

**Affiliations:** 1 Department of International Health, Care and Public Health Research Institute (CAPHRI), Maastricht University, 6200 MD Maastricht, The Netherlands; marcobarit99@gmail.com (M.B.); astghikbaghinyan@gmail.com (A.B.); lichitaindra.work@gmail.com (L.I.); kasia.czabanowska@maastrichtuniversity.nl (K.C.); 2 School of Medicine and Population Health, University of Sheffield, Sheffield S10 2TN, UK; 3 Communicable Disease Surveillance Centre (CDSC), Public Health Wales NHS Trust, Cardiff CF10 4BZ, UK; behrooz.behbod2@wales.nhs.uk (B.B.); christopher.roberts2@wales.nhs.uk (C.R.)

**Keywords:** field epidemiology, environmental health, climate change, expert interviews

## Abstract

Climate change and environmental degradation pose significant challenges to public health globally, intensifying the frequency and severity of related health incidents. Field epidemiology, traditionally focused on infectious disease outbreaks, is now increasingly recognised as vital in addressing environmental and climate-related health threats. This study explores how organisations like Public Health Wales (PHW) can develop field epidemiology services responsive to these emerging challenges. Semi-structured interviews were conducted with 18 global and national experts in field epidemiology, environmental health, and related disciplines. An inductive content analysis approach was used to identify themes relating to best practices, challenges, competencies, and future directions in environmental field epidemiology. Key findings emphasise the necessity for integrated, multi-sectoral collaboration, capacity building in One Health competencies, and innovative surveillance systems that incorporate environmental and climate data and exposure measures. Participants highlighted barriers such as fragmented governance, data quality issues, and resource constraints. The evolving role of field epidemiology includes applications of environmental and climate-related health phenomena to encompass prevention, preparedness, and recovery phases, supported by technological advancements and holistic health security frameworks. To effectively address environmental and climate-related health incidents, field epidemiology services must evolve towards integrated, multidisciplinary, and adaptive frameworks. Organisations like PHW may consider strengthening international collaboration, investing in workforce development, and implementing integrated surveillance systems that incorporate environmental drivers of health. These strategic priorities align with global public health functions and support resilient health systems capable of mitigating climate-related health risks.

## 1. Background

The severity, frequency, and adverse health outcomes of environmental incidents have been exacerbated by climate change [1]. Recognised as one of the most significant global health challenges of the 21st century, climate change drives numerous health-related consequences through rising temperatures, extreme weather events, shifting disease patterns, and environmental degradation [2,3,4,5]. These changes impact human health both directly through heat stress, injury, or illness during extreme events and indirectly, at population levels, via altered infectious-disease dynamics, food and water insecurity, and degraded living environments [6].

Environmental determinants account for nearly one-quarter of all global deaths [7]. Air pollution alone contributes to an estimated 7.4 million deaths annually, 3.2 million from household exposures and 4.2 million from ambient particulate matter [8]. Contaminated water, inadequate sanitation, occupational hazards, chemical exposures, and radiation further amplify this burden. Climate-driven extremes, including floods, droughts, and heatwaves, undermine food and water security and heighten the risks of malnutrition, vector-borne diseases, and heat-related mortality, projected to cause an additional 250,000 deaths per year between 2030 and 2050, with economic impacts of USD 2–4 billion annually by 2030 [9].

Wales faces comparable challenges, with Health Impact Assessments identifying coastal and rural communities at highest flood risk, while urban areas experience increasing heat-related illness [10,11]. A recent Public Health Wales (PHW) survey found that 20 percent of respondents aged ≥ 16 years approximately 516,471 individuals reported health issues linked to extreme weather [12]. Long-term exposure to air pollution in Wales is estimated to cause 1000–1400 deaths annually [13,14]. PHW has established monitoring and response frameworks for water contamination, chemical hazards (e.g., lead, asbestos, carbon monoxide), legacy land pollutants, noise pollution, and biological hazards such as harmful algal blooms [15,16,17]. These environmental exposures contribute substantially to respiratory, cardiovascular, gastrointestinal, and chronic diseases [18].

Field epidemiology applies rapid, on-site investigative methods to public health incidents, prioritising timely data collection, real-time risk assessment, and immediate intervention [19,20]. Traditionally focused on infectious-disease outbreaks such as the 1976 Legionnaires’ disease event and the 2014 West African Ebola epidemic, field epidemiology relies on descriptive studies to generate hypotheses for urgent policy and practice decisions [21,22,23]. There is growing recognition of its utility beyond outbreaks, to include humanitarian crises, chronic non-communicable conditions, and ‘wicked problems’ at the human–animal–environment interface [24,25]. Holistic approaches such as One Health framework underscore interconnectedness of ecosystems and support preventive measures against shared risks across sectors by utilising all available resources effectively [26]. These public health functions are intended to be equitable, proactive, population-centred, and sustainable, and ensure transparent use of available resources.

The COVID-19 pandemic further highlighted gaps in data availability, interdisciplinary collaboration, and equitable response systems, accelerating innovations in electronic data collection, genomics, bioinformatics, and wastewater surveillance [27,28]. Yet, traditional methods like direct observation, qualitative insights, descriptive analytics remain vital for actionable insights. Modern field epidemiologists require expanded competencies in data science, policy, and risk communication [29]. Despite successes such as Environmental Public Health Tracking (EPHT) frameworks that translate complex environmental and health data into stakeholder-tailored interventions [30] and evidence of epidemiology’s role in hazard evaluation and policy development [31], systematic research on deploying field epidemiology to address environmental health and climate challenges is scarce and often confined to practical implementation manuals and grey literature such as the Competencies for One Health Field Epidemiology (COHFE) framework [32]. Field epidemiology is useful and applicable in broad settings. However, its functions in addressing climate and environmental health incidents are not systematically evident in literature except for cases when proven successful.

Several jurisdictions have implemented practical climate–health integration strategies that inform this study’s recommendations. In the United States, CDC’s Climate-Ready States and Cities Initiative (CRSCI) supports state/local health departments to co-produce climate–health risk assessments and operational plans with partners (e.g., meteorological services, emergency management), while the National Environmental Public Health Tracking Network integrates environmental and health datasets to drive local action [33,34]. Across Europe, heat–health action plans (HHAPs) provide a structured model that links meteorological forecasts, graded heat alerts, and predefined public-health and social-care responses; in England, the UKHSA–Met Office Heat-Health Alerts are tied to surveillance and operational guidance and have been updated and used in recent seasons [35]. In the WHO African Region, Integrated Disease Surveillance and Response (IDSR) provides a unifying framework that links multi-hazard surveillance with predefined response actions across levels of the health system, illustrating how integration can be institutionalised in resource-constrained settings [36].

To understand how organisations like PHW can best develop field epidemiology services for climate change and related environmental health incidents, the aim of this study was to generate actionable insights that could inform the development of field epidemiology services in response to environmental and climate-related public health challenges. Specifically, the study sought to (1) identify current field epidemiology practices relevant to environmental and climate-related incidents through a rapid evidence review; (2) explore international best practices and internal expert perspectives on the effective delivery of these services; and (3) develop evidence-based recommendations to support organisations like PHW in establishing a comprehensive, responsive, and future-ready field epidemiology framework for environmental health emergencies. The World Health Organization’s (WHO) 2024 framework on Essential Public Health Functions (EPHFs), which defines twelve fundamental activities that form the backbone of comprehensive, integrated, and sustainable public health systems globally [37], was used as a guiding framework to critically analyse the findings.

## 2. Methods

The methodological foundation of this study draws upon four complementary frameworks to ensure conceptual clarity, methodological rigour and analytical depth. These include: (1) Conventional Content Analysis by Hsieh & Shannon (2005) [38], which has informed the study design to be inductive content analysis; (2) the World Health Organization (WHO) Stakeholder Mapping Guide [39], which guided the identification of experts based on levels of engagement and influence; (3) the Semi-Structured Interview Development Framework by Kallio et al. (2016) [40], which provided a structured, phased approach to developing the interview guide; and (4) the Qualitative Content Analysis Model by Elo and Kyngäs (2008), which informed the systematic organisation, grouping, and abstraction of qualitative data [41]. Inductive content analysis was chosen because prior scholarship on how field epidemiology services should be developed for environmental and climate-related incidents is limited. Our objective was therefore exploratory: to map the range of practices, barriers, and competencies reported by experts and to generate concepts and actionable propositions rather than test pre-specified hypotheses. This orientation justified open-ended interviewing, purposive sampling to maximise variation, and iterative coding until thematic saturation. Given the novel focus on field epidemiology in the context of climate and environmental incidents and lack of literature, this method was deemed especially suitable.

To ensure diverse and relevant perspectives, a total of n = 56 potential expert participants were identified through: (1) convenience sampling using internal and external stakeholder networks within PHW, (2) snowball sampling initiated by early informants, and (3) authorship of relevant peer-reviewed and grey literature in environmental health, field epidemiology, and public health service delivery. These experts were selected using the WHO Stakeholder Mapping methodology, ensuring representation across various levels of engagement and influence.

All potential participants received a formal invitation via email (template provided in File S1 in Supplementary Materials), which outlined the study’s aims, methodology, inclusion and exclusion criteria (File S2 in Supplementary Materials), and ethical safeguards. Participants were asked to review and sign an informed consent form prior to scheduling an interview. No communication was established with participants prior to study by the principal researcher. Of the 56 contacted, *n* = 13 initially agreed to participate, *n* = 2 declined due to time constraints, and *n* = 7 additional participants responded positively following a follow-up email sent one week later. Of these, *n* = 2 did not proceed with scheduling, resulting in a final sample of *n* = 18 expert interviews. This sample size was determined sufficient based on the principle of thematic saturation according to Guest et al. (2006) [42].

The semi-structured interview guide that can be found as File S3 in Supplementary Materials was developed using the framework by Kallio et al. (2016), encompassing the identification of theoretical prerequisites, integration of existing knowledge, pilot testing, and iterative refinement [40]. The interviews were conducted virtually via Google Meet by the principal researcher (25 years old, Türkiye born, male, master student with Public Health background) and the participant alone, audio-recorded with consent, and transcribed verbatim using recording by the interviewer. One interview cut in half and repeat interview was carried out due to an emergency of the participating expert. Topics included operational best practices, competencies, barriers, and recommendations for implementing field epidemiology services in response to environmental and climate-related public health threats.

The data analysis followed the inductive content analysis process outlined by Elo and Kyngäs (2008), comprising the preparation, organising, and reporting phases [41]. During the preparation phase, each complete interview transcript was treated as the unit of analysis to preserve contextual integrity. Transcripts were read repeatedly to achieve immersion and to gain a holistic understanding of the content. In the organising phase, open coding was conducted using NVivo (Release 1.7.2) software by a single coder (principal researcher). Notes and headings were recorded in the margins to capture key ideas, which were then systematically transferred into coding sheets. These codes were grouped based on similarity to form subcategories, which were further abstracted into generic and main categories through iterative comparison and interpretation. In the reporting phase, these categories were refined and interpreted in alignment with the study objectives. This process enabled the development of a coherent thematic structure that captured expert perspectives on the implementation of field epidemiology in environmental and climate-related health contexts.

To enhance methodological validity and reliability, three strategies were employed: member checking, triangulation, and peer debriefing. Member checking was conducted by sharing interview summaries with participants to validate the accuracy of interpretations [43]. Triangulation was achieved by integrating findings from the narrative literature review and interview findings in Section 4 [44]. Peer debriefing was facilitated through engagement with the Maastricht University Governance and Leadership in European Public Health (GLEPH) thesis group. The peer group consisted of two researchers apart from the main researcher (25-year-old Italy-born male MSc student with a background in medical anthropology and a 25-year-old Armenia-born female expert in public health). Their external perspectives provided critical reflection on the coding process, thematic structure, and interpretation of findings, strengthening the analytical rigor and transparency of the research process.

Finally, the COREQ (Consolidated Criteria for Reporting Qualitative Research) checklist that was adopted by Tong et al. (2007) can be found as File S4 in Supplementary Materials and was used as a guiding framework to ensure comprehensive documentation of researcher reflexivity, sampling rationale, data collection procedures, and analytical strategies [45].

## 3. Results

The characteristics of the 18 expert participants were presented as File S5 in Supplementary Materials in detail. Participants were evenly split between females (n = 9) and males (n = 9). Most participants were based in Europe (n = 13; 72%), with others located in North America (n = 1) and international institutions with global operational scope (n = 4). Participants represented a range of institutional affiliations, including public health institutes (n = 11), intergovernmental organisations (n = 5), academia (n = 1) and government (n = 1). Participants comprised both PHW staff (internal) and non-PHW experts (external). In total, 5/18 (28%) were Internal (PHW) and 13/18 (72%) were External.

Participants’ professional backgrounds included environmental epidemiology (n = 2), field epidemiology/One Health (n = 4), public health (including population health, health protection/health improvement, surveillance/epidemiology, statistics/methods; n = 7), environmental health/environment (n = 3), emergency preparedness and response (n = 1), and veterinary epidemiology (n = 1). Years of experience ranged from 6 to 41 (median 20). Interview durations ranged from 30 to 106 min (median ≈51 min)

Twenty-two sub-themes were developed and applied 446 times across the 18 interview transcripts. These sub-themes were grouped into seven overarching themes. Each code represented a unique instance where an idea was expressed, and while a single excerpt could be assigned multiple codes, repeated application was minimised through double-checking of overlaps. File S6 in Supplementary Materials represents the word cloud of the interview transcripts. The structure of coding was guided by inductive content analysis and refined through iterative interpretation and expert validation. While Table 1 presents the themes identified in alphabetic order, the detailed codebook export can be found as File S7 in Supplementary Material.

### 3.1. Theme 1: Best Practices in Environmental and Climate Health Field Work

This theme explores effective methods and approaches for applying field epidemiology in environmental and climate contexts. It includes four sub-themes: One Health Integration, Proof-of-Concept Pilots, Rapid, Evidence-Based Assessments, and Use of Established Tools.

A recurrent theme across interviews was the necessity for interdisciplinary collaboration through One Health Approach. Experts emphasised working jointly with animal, human, and environmental health sectors. An interdisciplinary approach also occurred in theme 7, but not necessarily under the One Health scheme.

“*With the Competencies for One Health Field Epidemiology (COHFE) framework, we said clearly this isn’t just about human health… it’s also about the health of animals and the environment.*”

(Participant ID #6)

“*Leptospirosis in Thailand… required joint animal-human-environment field investigations around water contamination.*”

(Participant ID #13)

Many participants described small-scale pilot projects as instrumental in demonstrating the utility of field epidemiology methods in non-traditional areas.

“*We developed pilot projects… carbon monoxide, chemicals in water, lead exposure in children… to demonstrate the service impact.*”

(Participant ID #9)

“*We developed forecasting models to estimate the impact of heatwaves and pollen spread… helpful in predicting health impacts.*”

(Participant ID #3)

Interviewees emphasised the importance of timely, actionable data collection to support immediate and short-term decision-making.

“*We go in the fourth week of training with real scoping missions… Fellows develop tools and collect field data… right on site.*”

(Participant ID #13)

“*Syndromic surveillance can detect real-time health spikes… NHS 111 calls, GP out-of-hours, admissions.*”

(Participant ID #1)

Established assessment frameworks such as, but not limited to, the Community Assessment for Public Health Emergency Response (CASPER) and the Public Health Situation Analysis (PHSA) were seen effective for environmental health incidents.

“*Wastewater and chemical surveillance are part of incident systems; integrating those with field methods is valuable.*”

(Participant ID #7)

“*We’re trying to build a climate change and health surveillance system for Wales… and align internationally.*”

(Participant ID #2)

### 3.2. Theme 2: Common Challenges and Barriers

This theme outlines systemic, operational, and epistemological barriers in adapting field epidemiology to environmental contexts. The sub-themes include Attribution & Complexity, Communication Barriers and Public Trust, Data Quality and Bias, Resource Constraints and Structural Barriers, and Siloed Mandates & Expertise Gaps.

Multiple participants emphasised the scientific and statistical difficulty of linking environmental exposures to specific health outcomes.

“*Attribution to climate change… is not always essential, but it’s a major evidentiary challenge.*”

(Participant ID #4)

“*Climate-linked diseases are hard to pin down statistically… links exist, but most effects are small and require huge studies to prove.*”

(Participant ID #17)

Challenges in public communication and building trust were commonly identified as barriers to effective fieldwork.

“*Some NGOs criticised our results… we couldn’t definitively state that incinerators were the sole cause… but our scientific approach showed a clear association.*”

(Participant ID #3)

“*People don’t understand why we act during uncertainty. If you say, ‘don’t drink the water or you’ll die,’ you lose trust.*”

(Participant ID #18)

Issues around retrospective data collection, underreporting, and lack of data integration were raised as concerns.

“*Most research is retrospective… it’s done months after the event, so it can’t guide response in real time.*”

(Participant ID #1)

“*We need to get better at learning from incidents… coding and analysing debriefs could help us see broader patterns.*”

(Participant ID #2)

Interviewees repeatedly mentioned that limited resources, under-recognition of environmental field epidemiology, and lack of dedicated staffing were key structural barriers.

“*Field epi is not always a recognised career. People return from training and can’t apply what they’ve learned.*”

(Participant ID #6)

“*Given the geopolitical space… field epidemiologists get pulled into international crises, drawing away from local capacity.*”

(Participant ID #2)

Many experts reported on the fragmentation between public health, environmental agencies, and emergency responders.

“*Social science expertise is often missing… needed to understand mental health impacts of long-term exposures.*”

(Participant ID #5)

“*There were no units specifically focused on environmental epidemiology… I was the first to introduce this focus in my region.*”

(Participant ID #3)

### 3.3. Theme 3: Equity, Vulnerable Populations & Risk Communication

This theme reflects the concern that standard public health approaches often fail to reach marginalised and high-risk populations. The sub-themes include Community Participation and Empowerment, Identify High-Risk Groups, and Tailored Communication & Trust Building.

Several participants highlighted the role of community involvement in ensuring more relevant and inclusive investigations.

“*Citizen science… where communities are involved in the research process from the beginning.*”

(Participant ID #3)

There was wide agreement that services should not overlook certain populations, and field epidemiology could help fill these gaps.

“*The Roma Traveller communities… tend to live in poor environmental conditions… field epi could help identify them.*”

(Participant ID #2)

“*Train frontline public health workers or GPs in basic field epi to ensure they can identify and support the most vulnerable.*”

(Participant ID #6)

Participants underlined the importance of adapting communication strategies to community needs and cultural contexts. This has appeared also as a challenge as well as a recommendation.

“*We must ensure transparency and work with the community to understand their concerns and needs.*”

(Participant ID #3)

“*Built relationships of trust and engagement in advance, vital when responding to outbreaks or investigations.*”

(Participant ID #4)

### 3.4. Theme 4: Essential Skills & Competencies

This theme focuses on the capabilities required for delivering effective field epidemiology in the context of environmental and climate-related health events. Experts emphasised that technical competence alone is insufficient. Instead, field epidemiologists must possess interpersonal, cross-sectoral, and adaptive skills. The sub-themes identified are: One Health Competences, Soft Skills, and Technical and Operational Skills.

Experts emphasised the growing necessity for field epidemiologists to be fluent in One Health thinking and capable of working across traditional disciplinary boundaries.

“*We defined shared and sector-specific competencies in the COHFE framework for human, animal, and environmental health.*”

(Participant ID #6)

“*Environmental health professionals understand legislation, regulations, and determinants of exposure.*”

(Participant ID #9)

Often referred to as “non-technical” abilities, soft skills, particularly communication, cultural awareness, and empathy, were described by many participants as indispensable.

“*Communication is considered a soft skill, but it’s not, it’s hard. Field epi must translate science into actionably simple messages.*”

(Participant ID #17)

“*We call them soft skills, but they’re the hardest. Without trust and communication, fieldwork fails.*”

(Participant ID #5)

Participants highlighted that field teams must be equipped with a mix of data-driven, analytical, and real-world operational skills from designing rapid surveys to understanding GIS and biosafety.

“*Sample collection, biosafety, and data interpretation are often missing or undervalued.*”

(Participant ID #18)

“*We train people in real-time outbreak management, logistics, and stakeholder engagement.*”

(Participant ID #13)

### 3.5. Theme 5: Expanding the Role of Field Epidemiology Beyond Outbreaks

A major finding of the study was the widespread agreement that field epidemiology could be more often applied to environmental and climate-related public health incidents. Experts argued that its scope should include preparedness, recovery, and long-term health monitoring. The two sub-themes here are: Application in Environmental and Climate Events and Support During Recovery Phase.

Experts explained that the toolkit of field epidemiology including rapid assessments, descriptive analysis, and community surveys is directly applicable to environmental exposures such as floods, wildfires, air pollution, and heatwaves. As these incidents are likely to occur more often with climate change, there are potential benefits in building field-service capacity.

“*Field epidemiology totally applies… the methods are helpful for readiness, integrating pieces of the puzzle, not just for outbreak investigation.*”

(Participant ID #10)

“*Think of the oil spill in Galicia… surveillance of health impacts, including mental health in volunteers and coastal communities.*”.

(Participant ID #15)

Multiple participants highlighted that field epidemiology is not only about acute response but also about informing longer-term interventions, system improvements, and community resilience.

“*I see field epidemiology as being more valuable in the latter stages of response into recovery.*”

(Participant ID #8)

“*Field epidemiology should be there throughout long-running incidents… it will inform the evolution of your response.*”

(Participant ID #5)

### 3.6. Theme 6: Future Directions

This theme captures forward-looking insights from participants about how field epidemiology must evolve in the face of increasingly complex and unpredictable environmental health threats. Sub-themes include Climate Modelling in Epidemiology, Holistic Health Security, and Technological & Methodological Innovation.

Several experts emphasised the importance of incorporating climate forecasting and predictive models into epidemiological practices. They argued that environmental field epidemiology must shift from reaction to prevention.

“*We must predict when landslides or floods will occur and know what diseases will follow… this is how we make epi proactive.*”

(Participant ID #6)

“*Modelling health service use during climate events… predictive planning is where field epi adds major value.*”

(Participant ID #7)

A number of participants advocated for a more integrated, systems-based approach that situates field epidemiology within broader national and international health security frameworks.

“*We must treat prevention as equal to cure… technological advances and field epi must merge to stop disease before it spreads.*”

(Participant ID #6)

“*We should move from planning for the minimum to planning for the reasonable worst-case scenario.*”

(Participant ID #8)

Several experts discussed the opportunities offered by digital innovation, mobile technology, AI, and novel surveillance tools. However, they emphasised that such advances must be implemented with caution and grounded in strong field methodology.

“*AI, wastewater surveillance, remote sensing… these tools are here. We need field epi to make them actionable.*”

(Participant ID #13)

“*We should be forecasting health impacts of weather events… not reacting after admissions spike.*”

(Participant ID #11)

### 3.7. Theme 7: Intersectoral Collaboration Is Critical

All participants strongly emphasised the necessity of coordinated action across sectors and agencies to address environmental and climate-related health threats. Effective field epidemiology requires engagement from public health, environmental, emergency services, meteorology, and international partners. Two sub-themes were identified: Global & Cross-Agency Partnerships and Local & National Coordination.

International collaboration was seen as essential for knowledge exchange, capacity-building, and standard-setting. Participants referenced successful examples from the U.S., Europe, Africa, and international One Health networks.

“*We work with environmental agencies, the European Commission, and WHO… benchmarking and avoiding duplication are critical.*”

(Participant ID #10)

“*You need multi-sector consortia… health professionals must work with agriculture, engineering, water infrastructure sectors to identify options, not just evaluate impacts.*”

(Participant ID #13)

“*Good investigation requires hydrogeologists, atmospheric scientists, medical anthropologists… beyond health.*”

(Participant ID #17)

On the domestic side, respondents stressed the value of multi-agency integration, particularly between environmental health teams, health protection, emergency responders, and community actors.

“*Response centres need clear task forces… daily meetings with assigned roles across agencies.*”

(Participant ID #13)

“*We always work with Natural Resources Wales, local authorities, and others in writing flood mitigation plans.*”

(Participant ID #8)

## 4. Discussion

This study’s thematic findings strongly align with the WHO’s EPHFs which encompass a broad spectrum from surveillance, emergency management, health protection, disease prevention, health promotion, to workforce development and multisectoral coordination. Hence, these thematic findings offer a practical lens through which these high-level functions can be operationalised, particularly in the context of environmental and climate change-related health incidents. Although themes were presented alphabetically in the Results, they are discussed here in order of coding frequency.

### 4.1. Common Challenges & Barriers

#### 4.1.1. Siloed Mandates & Expertise Gaps

A key challenge identified by participants is the existence of siloed mandates both within public health sectors (e.g., environmental health, surveillance, emergency response) and between sectors critical for climate change mitigation and adaptation and response, such as agriculture, housing, and transportation. These silos limit communication and coordination, causing fragmented efforts in health services and systems. For example, different agencies hold data that is rarely integrated, impeding timely and comprehensive responses. Alongside structural silos, there are notable expertise gaps in understanding the complex intersection of environment and health [46]. Field epidemiology service can play a bridging role—brokering pre-agreed roles, data-sharing, and joint tasking between local agencies while linking local operations to national decision-making.

#### 4.1.2. Attribution and Complexity

Attributing health impacts to climate change is complex due to multiple interacting factors influencing outcomes. Experts highlighted that the challenge lies in untangling the effects of climate-related environmental exposures from other social and ecological influences.

Current limitations in data quality, spatial coverage, and temporal resolution hinder accurate attribution. Ebi et al. have highlighted successful attributions in linking heatwave deaths, vector-borne diseases like Lyme, or waterborne illnesses such as Vibrio infections to climate change by utilising detailed, long-term data across health, environment, and climate domains [47].

#### 4.1.3. Data Quality and Bias

There is a potential that much of the available health data is retrospective, incomplete, and inconsistently collected across different regions and systems, which limits the ability to detect emerging trends promptly and to respond proactively. Underreporting and variations in data collection methods further reduce the accuracy and reliability of information. Limited integration of qualitative and quantitative data between actors and with behavioural and social science insights constrains a comprehensive understanding of the multifaceted drivers behind health outcomes. Behbod et al. have highlighted in an international comparison of health registers the need for early, standardised, and systematic data collection to support timely epidemiological assessments and follow-up through health registers, showing that pre-established protocols improve data quality, reduce bias, and minimise loss to follow-up [48].

#### 4.1.4. Resource Constraints, and Structural Barriers

Health systems worldwide face significant resource constraints that hinder their capacity to respond effectively to environmental health risks. The Lancet Countdown 2022 report highlights that the COVID-19 pandemic worsened these challenges, with 30% of cities reporting reductions in funding for climate action as a direct result of the pandemic. Resource and structural constraints disproportionately affect low- and middle-income countries, exacerbating existing inequities and undermining global health security [49].

### 4.2. Expanding the Role of Field Epidemiology Beyond Outbreaks

#### 4.2.1. Application in Environmental and Climate Events

Participants highlighted the proven success of field epidemiology techniques in various settings including humanitarian contexts and emphasised their potential critical role in responding to environmental and climate-related health events.

According to Frérot et al. (2018), modern epidemiology has expanded beyond infectious diseases to include the study of health phenomena broadly, shifting from a narrow disease-centred definition toward a more holistic approach that incorporates health impacts from environmental and social determinants [50]. Expanded applicability of field epidemiology is also closely linked with public health messaging, and behavioural change among community.

#### 4.2.2. Support During Recovery Phases

Recovery involves addressing long-term health impacts, including mental health consequences, that often persist well beyond the acute incident. Field epidemiologists can also contribute to understanding the broader public health consequences of recovery actions, such as displacement, insurance issues, and access to essential services, identify unmet needs and recovery trajectories. The recovery phase offers opportunities for learning through debriefing and horizon scanning to improve preparedness for future events. This advances the literature by specifying recovery-phase field work—monitoring mental-health and social impacts, establishing follow-up registers, and closing the loop between findings and service access—rather than focusing solely on acute response.

### 4.3. Best Practices in Environmental & Climate Field Work

#### 4.3.1. One Health Integration

Participants highlighted the vital role of One Health integration in field epidemiology, emphasising the close links between human, animal, and environmental health amid climate challenges. Field epidemiology supports this by investigating health risks crossing species and ecosystems. Examples include deforestation increasing zoonotic disease exposure, hikers in Wales encountering infected animals, and poultry farming where antimicrobial-resistant pathogens spread through communities, food supplies, and the environment. Rising vector-borne diseases due to warmer temperatures further demand collaboration among public health, veterinary, and environmental experts. Diseases in food animals also threaten livelihoods and economies, requiring coordinated responses [51]. Our findings complement competency frameworks by showing One Health collaboration as routine practice at different levels, not an exceptional add-on.

#### 4.3.2. Proof-of-Concept Pilots

Informants emphasised the importance of transferring innovative models that have proven effective in other settings, while also taking into account system readiness criteria as defined by Schloemer et al. [52], and applying insights on diffusion and adoption processes including understanding adopters and system antecedents for successful scale-up as demonstrated by Greenhalgh et al. [53].

#### 4.3.3. Rapid, Evidence-Based Assessments

Participants highlighted the value of real time data analysis and data sources such as syndromic data, emergency calls, hospital admissions and even search trends which help with identification and resource allocation. However, they also noted challenges like data delays and potential biases in rapid assessments.

The WHO guidelines emphasise that hazard characterisation and impact assessments whether rapid or not should systematically evaluate epidemiological evidence with clear exposure definitions, health outcomes, and transparent communication [54].

#### 4.3.4. Use of Established Tools

Traditional two-by-two tables are insufficient for the broad scope of modern field epidemiology. Participants identified tools like early warning systems, wastewater surveillance, PHSA and CASPER as valuable for systematic data collection and analysis to assess health needs, support planning, and guide public health responses. Briggs discusses added value of other methodologies such as Health Impact Assessment (HIA) and Comparative Risk Assessment (CRA) allowing for investigating complex risks, set within wider social, economic and environmental contexts [55].

### 4.4. Intersectoral Collaboration Is Critical

#### 4.4.1. Global & Cross-Agency Partnerships

Effective response requires coordinated planning and action across health, agriculture, engineering, water infrastructure, and other sectors to not only evaluate impacts but to actively identify and implement solutions [56] as well as international practices and global learning. The CDC Climate-Ready States and Cities Initiative (CRSCI) demonstrates how cross-sector and cross-agency collaboration through technical partnerships, knowledge sharing, and networked learning, can help integrate health into local and national climate response strategies, building adaptive capacity and disseminating best practices widely [57].

The findings have underscored cross-sectoral collaboration, multiagency coordination, interprofessional training and learning, one health integration under different themes and these results may be read as the same, however they have differences in terms of their specific focus, scope, and operational implications. While cross-agency collaboration emphasises strategic partnerships among diverse sectors (e.g., agriculture, emergency services, health), multiagency coordination highlights operational synchronisation among distinct agencies or organisations to ensure timely responses. Interprofessional training and learning specifically target skill development and capacity-building across professions and disciplines, fostering mutual understanding and shared competencies. In contrast, One Health integration explicitly incorporates a holistic conceptual framework that links human, animal, and environmental health, guiding collaborative actions toward unified health outcomes.

#### 4.4.2. Local and National Coordination

A multi-agency approach is essential at all governance levels such as district, county, nation, and country, for effective planning and response. McCulloh et al. emphasise that local agencies, supported by national bodies and global learning, serve as the backbone of public health preparedness, yet can struggle without clear integration into broader frameworks [58]. In the UK context, this translates to coordinated collaboration among agencies in Scotland, England, Wales, and Northern Ireland, and alignment with the UK Health Security Agency (UKHSA). Participants noted that without systematic, multi-agency coordination across these layers, responses risk fragmentation and inefficiency. Strengthening partnerships, data sharing, and joint planning at each level is necessary to build resilient health systems, aligning with WHO’s Essential Public Health Functions, which call for the involvement of stakeholders from air, water, ecological, and food systems in decision-making related to health and climate change.

### 4.5. Essential Skills & Competencies

#### 4.5.1. Soft Skills

Although often viewed as secondary to technical expertise, participants consistently emphasised that soft skills particularly communication, empathy, cultural competence, and adaptability are indispensable for effective field epidemiology, especially in crisis or community-facing roles. Communication, in particular, emerged not only as an essential skill but also as an independent sub-theme, highlighting its multifaceted importance. Participants distinguished between communication as a core competency and its application within the broader domains of fieldwork. This marks a significant shift from perceiving field epidemiology as a purely technical discipline to recognising the essential contribution of interpersonal effectiveness to public health outcomes. Moreover, participants noted that strong communication skills enable field epidemiologists to support behavioural change and deliver clear, trustworthy public health messages capabilities that are especially critical in engaging communities and fostering resilience during emergencies, as Whiley et al. (2023) document the use of Environmental Health Officers in South Australia for education, communication, and compliance monitoring during the COVID-19 response [59].

#### 4.5.2. One Health Competences

One Health which is the recognition that human, animal, and environmental health are deeply interconnected, emerged as a central theme in this study. Participants specifically referenced the COHFE framework as a valuable guide for building the multidisciplinary skills required in today’s complex health landscape [32]. The framework emphasises systems thinking, multisectoral collaboration, and integrated surveillance, enabling field epidemiologists to account for how environmental changes such as extreme weather, pollution, and biodiversity loss shape disease emergence and health outcomes across species. COHFE’s essential skills include collaboration and partnership, systems thinking, leadership, and clear communication with diverse stakeholders. It also prioritises technical competencies such as integrated surveillance and data management, field investigations, risk assessment, laboratory and diagnostic coordination, and environmental and ecological assessment.

#### 4.5.3. Technical and Operational Skills

Technical and operational skills are foundational to effective field epidemiology in environmental and climate health contexts. Participants consistently noted the importance of rapid data collection, advanced data management, and the application of tools such as GIS, environmental surveillance, and statistical modelling. Skills in survey design, exposure assessment, and linking complex environmental data to health outcomes are increasingly critical as the scope of field epidemiology expands beyond infectious diseases. Operational readiness including the ability to deploy quickly, use specialised equipment, and maintain methodological rigour even in fast-moving scenarios was frequently highlighted. As environmental hazards become more frequent and multifactorial, the capacity to adapt classical epidemiological methods, ensure data quality under time pressure, and integrate new forms of surveillance will be essential for timely, evidence-based public health action. Leonardi et al. have highlighted the indispensable role of capacity building and training to acquire relevant competencies for pressing issues in environmental field work such as pollution, biodiversity loss, and climate change. This includes integrating environmental public health principles into existing curricula for public health professionals, clinicians, and allied sectors, fostering ecological sustainability, and promoting collaborative approaches across disciplines to enhance community resilience and public health outcomes [60].

### 4.6. Equity, Vulnerable Populations & Risk Communication

#### 4.6.1. Community Participation and Empowerment

Participants emphasised the inherent community proximity of field epidemiology, highlighting its frontline role like general practitioners or community nurses. Field epidemiologists, by virtue of their direct presence in the community, are well-positioned to facilitate active participation and empowerment. Engaging communities directly in investigations ensures interventions are relevant, culturally appropriate, and effective. Interaction with community and making their voiced heard, not only improves data quality by adding a qualitative value to it but also fosters local ownership, trust, and sustained behavioural change, strengthening overall public health resilience during environmental and climate health events.

#### 4.6.2. Identify High-Risk Groups

Interviewees consistently emphasised the critical importance of proactively identifying and reaching vulnerable groups, including the elderly, displaced individuals, marginalised communities, and populations disconnected from routine healthcare systems. Participants noted these groups are often underrepresented or invisible in standard surveillance data, requiring targeted outreach, robust mapping of local vulnerabilities, and partnerships with local organisations.

#### 4.6.3. Tailored Communication & Trust Building

While communication emerged as an essential skill under the broader “soft skills” theme, participants further emphasised its critical importance as a distinct domain of field epidemiology practice especially given the shift towards more community-facing roles. Tailored communication, characterised by cultural sensitivity, clarity, and trust-building, was identified as indispensable in effective public health responses to environmental and climate health incidents. Field epidemiologists increasingly deal with situations where environmental health incidents are directly linked to local community practices or behaviours. Participants cited examples, such as outbreaks linked to specific dietary practices (e.g., consumption of certain animals or culturally significant foods), illustrating the importance of understanding and addressing local behaviours sensitively. In these contexts, field epidemiology requires not only identifying the source of the outbreak but also carefully communicating with communities to alter risk behaviours without stigmatisation.

### 4.7. Future Directions

#### 4.7.1. Climate Modelling in Epidemiology

Participants acknowledged the critical role of climate modelling in enhancing field epidemiology’s capacity to predict and plan for health impacts under various climate scenarios. Integrating climate data with epidemiological surveillance improves anticipation of disease outbreaks and environmental health risks. The recent Lancet Planetary Health review highlights the evolving landscape of software tools designed specifically for climate-sensitive infectious disease modelling. It identifies a range of operationalised tools that incorporate climate inputs to forecast disease risk and support public health decision-making. However, the review also notes significant gaps, such as a shortage of tools for respiratory, food-borne, and water-borne diseases, and the underrepresentation of models tailored for emerging hotspots or non-endemic regions [61]. Participants reflected these challenges and more by emphasising the need for flexible, user-friendly modelling systems that can be adapted locally and integrated into routine surveillance of also non-infectious, temperature related diseases.

#### 4.7.2. Holistic Health Security

Environmental health issues have traditionally been managed by targeting individual pollutants or exposures. However, the complexity of today’s hazards exposes practical limitations in current tools. For respiratory risks, wildfire-smoke and heat models often use coarse exposure fields that miss street-level and indoor conditions, rarely assimilate near-real-time syndromic data (e.g., NHS 111, GP, ED) for rapid calibration, and struggle with compound exposures (heat, smoke, ozone/pollen), which blunts short-term forecasting of asthma/COPD exacerbations. For water-borne risks after flooding, sparse high-resolution hydrology and combined sewer overflow data, infrequent microbial sampling, limited coverage of private wells, and weak coupling to gastrointestinal syndromic indicators delay early warnings and targeted boil-water advice. More broadly, many climate-sensitive models are locally tuned with limited external validation, provide opaque or unquantified uncertainty, and under-represent non-infectious endpoints (e.g., heat-exacerbated cardiovascular disease). Effective solutions must operate across clearly defined scales, with actionable levers at each level: individual (clinical risk advice and self-protection behaviours), local/municipal (neighbourhood air-quality management and care-home heat plans), regional/national (integrated surveillance networks linking syndromic data with meteorology, air quality, hydrology and wastewater, alongside mutual-aid arrangements and policy triggers), and international (cross-border alert systems and shared data standards to detect transboundary hazards) [62]. Interviewees consistently emphasised the need for a systems-level approach that integrates surveillance, prevention, preparedness, and resilience across both health and environmental domains. Rather than focusing solely on health outcomes such as heat-related deaths, participants highlighted the importance of monitoring upstream drivers of illness.

As climate change intensifies, healthcare systems will face increasing demand and complexity. Participants stressed the necessity of horizon scanning and maintaining flexibility to respond to unpredictable climate impacts. Moving beyond minimal compliance, they advocated for planning based on reasonable worst-case scenarios and incorporating graded risk assessments that raise awareness and inform more robust, resilient public health strategies. Preventive resilience is being embedded in critical settings such as care homes, prisons, and schools that are sectors vital for societal functioning. There is growing recognition that real health security derives from prevention and infrastructure, not reactive measures alone. This includes addressing mental health and wellbeing.

Governance complexity and variable capacity across agencies and regions remain significant barriers to holistic health security. Participants and literature alike advocate for the integration of environmental health into broader emergency management frameworks, emphasising that all essential public health services—surveillance, preparedness, and local response—must function synergistically. Lauriola et al. reflected in their review these insights in practical applications worldwide, where EPHT programmes in countries such as the United States, England, France, Canada, and China holistically integrated data systems, surveillance, and local response, tracked environmental hazards, and measured exposures and health outcomes [30].

#### 4.7.3. Technological and Methodological Innovation

Participants specified tool categories for improved exposure modelling, including GIS and spatial analysis for risk mapping and hotspot detection; earth-observation/remote sensing inputs to fill spatial gaps; and process models such as air-pollutant dispersion/smoke transport and flood-risk/hydrological models. They also pointed to integrated data platforms that fuse syndromic and laboratory data, wastewater surveillance, low-cost air and water sensor networks/mobile monitoring, and environmental regulator feeds (air/water quality, combined sewer overflows). Several suggested cautious use of AI for anomaly detection, nowcasting, and data assimilation across these streams, with transparent validation and uncertainty reporting.

### 4.8. Limitations

This study is primarily focused on Public Health Wales (PHW), which may limit the generalisability of its findings to other public health organisations. While many insights are likely relevant to similar institutions, the degree to which these recommendations apply beyond PHW remains uncertain without further comparative research. The qualitative design, based on 18 expert interviews, provided rich contextual information and thematic saturation; however, the purposive and convenience sampling methods used may have introduced selection bias, potentially favouring participants already engaged with or supportive of PHW. The non-response bias also might have affected our results alongside with the participants being public sector dominant. The study also did not include quantitative data to measure the effectiveness or cost–benefit of field epidemiology interventions, limiting the ability to evaluate concrete outcomes. Furthermore, the complexity and multifaceted nature of environmental and climate health challenges, spanning local to global scales and diverse socio-political contexts, may mean that the study’s scope does not fully capture all relevant determinants and factors influencing public health responses.

## 5. Conclusions

The findings highlight the importance of translating national-level collaboration frameworks into effective local cooperation. While national bodies collaborate at strategic or policy levels, their local counterparts, such as community healthcare providers, emergency services, or environmental agencies, might lack established mechanisms for routine collaboration. Furthermore, certain domains may not have a clearly defined or dedicated presence at the local level. In this context, field epidemiology services can serve effectively as local extensions for domains traditionally lacking robust local structures, by providing integrated surveillance, assessment, and community engagement directly within affected areas. Field epidemiology thus acts as a critical bridging function within local contexts, facilitating coordination among fragmented local agencies and simultaneously connecting local operational realities with national decision-making processes

Therefore, there is a pressing need to advance integrated surveillance approaches that combine climate, environmental, and health data. While establishing comprehensive systems is a long-term endeavour, immediate focus on enhancing real-time monitoring tools—such as syndromic surveillance and environmental indicators—can improve preparedness and response. Additionally, cross-sector collaboration as both a short-term and a long-term goal remains critical. Public health bodies should actively promote partnerships involving healthcare providers, environmental experts, veterinarians, and community stakeholders. Regular communication and joint training support shared situational awareness and coordinated action.

Building workforce capacity through structured and continuous education—including simulations, mentorship, and multidisciplinary engagement—is fundamental. Training should be accessible not only to specialists but also to frontline workers who serve as vital links to communities. Systematic mapping of stakeholders and understanding region-specific needs help tailor services and optimise resource allocation. Recognising the diversity of ecological and social contexts ensures interventions are appropriately targeted. Addressing organisational and governance barriers is essential for effective multi-agency collaboration. Capacity in translating evidence into timely action, shared leadership, joint contingency planning, and mechanisms to overcome silos contribute to stronger integrated public health responses.

The envisioned field epidemiology service identified through this study would have a clear purpose of enhancing preparedness, rapid response, and long-term recovery from environmental health incidents, which is applicable not only in Wales, but also globally. It would leverage an integrated, multidisciplinary approach underpinned by the One Health framework, connecting human, animal, and environmental health sectors. The service would ideally comprise specialised staff trained in interdisciplinary methods, robust integrated surveillance and modelling systems combining health and environmental data, rapid assessment capabilities and targeted community engagement strategies to address vulnerabilities and enhance resilience.

## Figures and Tables

**Table 1:** Identified Themes and Sub-themes.

**Theme 1: Best Practices in Environmental & Climate Field Work**
Sub-theme: One Health Integration
Sub-theme: Proof-of-Concept Pilots
Sub-theme: Rapid, Evidence-Based Assessments
Sub-theme: Use of Established Tools
**Theme 2: Common Challenges & Barriers**
Sub-theme: Attribution & Complexity
Sub-theme: Communication Barriers and Public Trust
Sub-theme: Data Quality and Bias
Sub-theme: Resource Constraints and Structural Barriers
Sub-theme: Siloed Mandates & Expertise Gaps
**Theme 3: Equity, Vulnerable Populations & Risk Communication**
Sub-theme: Community Participation and Empowerment
Sub-theme: Identify High-Risk Groups
Sub-theme: Tailored Communication & Trust Building
**Theme 4: Essential Skills & Competencies**
Sub-theme: One Health Competences
Sub-theme: Soft Skills
Sub-theme: Technical and Operational Skills
**Theme 5: Expanding the Role of Field Epidemiology beyond Outbreaks**
Sub-theme: Application in Environmental and Climate Events
Sub-theme: Support During Recovery Phase
**Theme 6: Future Directions**
Sub-theme: Climate Modelling in Epidemiology
Sub-theme: Holistic Health Security
Sub-theme: Technological & Methodological Innovation
**Theme 7: Intersectoral Collaboration is Critical**
Global & Cross-Agency Partnerships
Local & National Coordination

## Data Availability

The datasets (i.e., interview transcripts and coding framework) generated and analysed during the current study are not publicly available due to confidentiality agreements and the inclusion of potentially identifiable expert perspectives. However, anonymised excerpts and thematic matrices are available from the corresponding author on reasonable request.

## Supplementary Materials

The following supporting information can be downloaded at: https://www.mdpi.com/article/10.3390/ijerph22091452/s1: File S1: Email draft sent to invite experts; File S2: Inclusion and Exclusion Criteria for the Study; File S3: Semi-structured validated interview guide; File S4:Consolidated Criteria for Reporting Qualitative Studies (COREQ) Checklist; File S5: Participant Demographics; File S6: Word Cloud of the interview transcripts; File S7: Detailed Codebook of the identified themes; File S8: Supplementary Quotations.
