# Supplementary material for: Role of Field Epidemiology in Environmental and Climate Change-Related Health Incidents in Wales: A Qualitative Analysis Through Expert Interviews
Source: Int J Environ Res Public Health. 2025 Sep 18;22(9):1452. doi: 10.3390/ijerph22091452 (PMC12469667; doi:10.3390/ijerph22091452)
Supplement: Supplementary file 1 [file ijerph-22-01452-s001.zip › ijerph-3833913-supplementary.pdf]

Supplementary Material

File S1: Email draft sent to invite experts

Dear (Interviewee),

I hope this email finds you well.

My name is Ömer Faruk Sönmez, and I am a master's student in Governance and Leadership in European Public Health at Maastricht University, conducting research in collaboration with Public Health Wales. My thesis explores the development of field epidemiology services to address environmental and climate change-related health incidents in Wales.

Given your expertise in this field, I would be honored to invite you to participate in a semi-structured interview to gain valuable insights into best practices and challenges in expanding field epidemiology services to enviromental health incidents. This interview will contribute to the following research question:

"How can Public Health Wales best respond to environmental and climate change-related incidents through the delivery of field epidemiology services?"

The interview will last 30-60 minutes and will be conducted via Google Meet. With your consent, it will be recorded for analysis. You can schedule a time that suits you directly via this booking link: (Booking Link)

Consent Form Requirement
For ethical compliance, I kindly ask you to review and sign the attached Informed Consent Form before the meeting. Please send the signed form to me at (interviewer email) prior to the interview. The consent form outlines all study details, including confidentiality, data protection, and your right to withdraw at any time.

If you have any questions or need further clarification, please don’t hesitate to reach out. Thank you for considering this invitation—your expertise is invaluable in shaping recommendations for Public Health Wales.

Looking forward to your response.

Best regards,

File S2: Inclusion and Exclusion Criteria for the Study

Inclusion Criteria	Exclusion Criteria
Participants with at least 3-5 years of professional experience in field epidemiology, public health, environmental health.	Participants who cannot commit to follow-up questions or clarification if needed.
Individuals affiliated with Public Health Wales or other public health agencies around the world with the relevant scope of work.	Participants whose primary expertise lies outside the scope of the study
Participants fluent in English for effective communication during interviews.	Participants bound by confidentiality agreements that prevent open sharing of relevant insights or data.
Participants who are willing to participate and can commit to scheduled interviews and the consent process.	Participants with potential conflicts of interest, such as ties to commercial entities related to the study scope.
Participants that have experience with international field epidemiology proffesional networks, academia and private sector that is concerned with field epidemiology and environmental health challenges.	If too many participants from one sector or institution are included, additional individuals from the same group may be excluded to maintain diversity.
Individuals who may have been directly involved in policy-making or implementing field epidemiology services.	

File S3: Semi-structured validated interview guide **Interview Guide**

A. Participant Information

**Participant No.:**	#
**Participant name & contact information:**	
**Participant Background & Education:**	
**Participant Institution & Expertise Area:**	
**Meeting Platform:**	
**Date:**	
**Interviewer/Note-taker:**	Ömer Faruk Sönmez

B. Interviewer Checklist

- Confirm the date and time and send a reminder to the participant before the meeting.
- Make sure you send the link for the online meeting to the participant.
- Ascertain the allotted interview time acceptable to the participant.
- Print a copy of the Interview Guide to take notes during interviews.
- Make sure participant has signed and agreed the informed consent form.

C. Introduction and Consent Statement

Say: Thank you for agreeing to participate in this interview. This study aims to assess how field epidemiology services can contribute to incident preparedness, resilience, and response in environmental and climate change-related health incidents, with a particular focus on Wales. Specifically, I am gathering insights from public health and environmental health experts, academics, field epidemiologists, Emergency Preparedness, Resilience and Response (EPRR) staff, and professionals from public health authorities and agencies. The objectives of this research are to: (1) identify the role and applicability of field epidemiology approaches in managing environmental health incidents; (2) examine best practices and challenges in integrating these approaches into existing public health systems; and (3) develop evidence-based recommendations for Public Health Wales to enhance their field epidemiology capacity. Your input will directly contribute to shaping a final report with actionable recommendations for service development.

I'm carrying out this research in an external placement in Public Health Wales in Cardiff, UK under the supervision of Prof.dr. Katarzyna Czabanowska internally from Maastricht University and Dr.Behrooz Behbod and Christopher Roberts from Public Health Wales.

Participant Consent Statement:

Say: Your input during this interview will be used in my master thesis at Maastricht Uiversity. We already asked you to sign informed consent form, I will begin the recording once the signed informed consent form has been delivered to me through email. You may also withdraw your responses from the interview at any time. Do you have any questions for me before that about the informed consent?

Ask: Do you agree for the data collected in the study to be used in the thesis?
☐ Yes ☐ No Notes:
Ask: Do you also agree to have the interview recorded?
☐ Yes ☐ No Notes:
Ask: Do you have any conflict of interest regarding this topic?
☐ Yes ☐ No Notes:
Ask: Do you have any questions before we proceed?
☐ Yes ☐ No Notes:

D. Interview Structure
Say: Our interview will consist of four parts. We will first begin by getting to know you, your research and proffessional expertise better, then we will talk about the field epidemiology services and its potential applications in environmental and climate change related incidents Then we will continue our talk with policies and practices regarding its development and draft reccomendations for Public Health Wales and reflections. Discussion regarding the policy and reccomendations of field epidemiology services constitutes the main aim of my thesis research.

E. Questions
The interview will be semi-structured and conducted online or in a private setting convenient for the participant. Participants will receive a unique link for their scheduled interview. The discussion will focus on professional insights rather than personal information, with the aim of understanding best practices and challenges in developing field epidemiology services for environmental health. Interviews will be recorded, anonymized during transcription, and analyzed using Atlas.ti. Participants can opt not to answer specific questions and can withdraw before publication. Follow-up questions may arise based on responses.

Interview Structure

Part 1: Professional Background
1. **Introduction and Expertise**

   Can you tell us a little about yourself and your work?

What is your role in dealing with environmental or climate-related issues, like floods, pollution, or other challenges in your job?

Part 2: Field Epidemiology Services

Say: Field epidemiology is like being a detective for health problems. When something like pollution, dirty water, or changes in the weather hurts people, field epidemiologists attempt to figure out what's wrong and provide this information in a timely manner. They ask questions, collect information, design studies to identify sources, and work with others to stop people from getting sick. By finding problems quickly, they help communities become safer and better prepared for the future. Their work also helps Inform action, including advocacy for evidence-based policy influence and change.

2. **Perceived Value of Field Epidemiology Services**

   What do you see as your role in relation to field epidemiology? Are you directly involved, benefiting from its insights, or working alongside field epidemiologists in a different capacity?

   Have you heard of field epidemiology before? Do you think it could help in tackling environmental or climate-related problems? If yes, how?

   What specific problems could field epidemiology help solve in your community or work?

3. **Best Practices and Challenges**

   Have you or your organisation worked with field epidemiologists to address environmental or climate-related issues? If yes, what methods or practices worked well?

   What challenges have you seen when trying to solve these problems?

   Can you see any situations where field epidemiology approaches lead to unintended consequences, such as confusion, adverse effects etc? Do you have any examples?

4. **Intersectoral Collaboration**

   Can you think of any good examples of teams or organisations working together successfully on these kinds of problems?

5. **Skills and Competencies**

   What skills or knowledge do you think field epidemiology teams should have in order to better respond environmental health incidents?

Part 3: Policy and Recommendations

6. **Policy and Strategic Recommendations**

   How do you think health organisations like Public Health Wales can better prepare for and respond to environmental problems?

   Do you think working with international teams or experts could improve efforts to tackle these issues?

7. **Equity and Access**
   How might a service like field epidemiology ensure all communities, including the most vulnerable, are supported?

   At what stages—before, during, or after environmental or climate-related incidents—do you think field epidemiology should play a role? Should its involvement differ across these phases, and if so, how?

Part 4: Reflections

8. **Future Directions**

   What do you think the future looks like for solving environmental health problems? Where should efforts focus over the next 10 years? Is there anything else you'd like to share about how field epidemiology could grow or improve in this area?

Closing

Say: Thank you for your participation. Your insights are invaluable to this research. If you have further thoughts or questions, feel free to contact me or the research supervisors. A summary of the findings will be shared with you before publication for your feedback.

File S4: Consolidated Criteria for Reporting Qualitative Studies (COREQ) Checklist

Developed from:

Tong A, Sainsbury P, Craig J. Consolidated criteria for reporting qualitative research (COREQ): a 32-item checklist for interviews and focus groups. International Journal for Quality in Health Care. 2007. Volume 19, Number 6: pp. 349 – 357.

No.	Item	Guide Questions / Description	Where is it reported
**Domain 1: Research Team and Reflexivity**			
**Personal Characteristics**			
**1**	Interviewer/facilitator	Which author/s conducted the interview or focus group?	File 3
**2**	Credentials	What were the researcher's credentials? (e.g., PhD, MD)	Methods Section
**3**	Occupation	What was their occupation at the time of the study?	File 1
**4**	Gender	Was the researcher male or female?	Methods Section
**5**	Experience and training	What experience or training did the researcher have?	Methods Section
**Relationship with Participants**			
**6**	Relationship established	Was a relationship established prior to study commencement?	Methods Section
**7**	Participant knowledge of the interviewer	What did the participants know about the researcher (e.g., personal goals, reasons for the research)?	File 1
**8**	Interviewer characteristics	What characteristics were reported about the interviewer/facilitator?	File 1
**Domain 2: Study Design**			
**Theoretical Framework**			
**9**	Methodological orientation and theory	What methodological orientation was stated to underpin the study? (e.g., grounded theory, phenomenology)	Methods Section
**Participant Selection**			
**10**	Sampling	How were participants selected? (e.g., purposive, snowball)	Methods Section
**11**	Method of approach	How were participants approached? (e.g., email, in person)	File 1
**12**	Sample size	How many participants were in the study?	File 5

**13**	Non-participation	How many refused to participate or dropped out? Reasons?	Methods Section
**Setting**			
**14**	Setting of data collection	Where was the data collected? (e.g., clinic, home)	Methods Section
**15**	Presence of non-participants	Was anyone else present besides participants and researchers?	Methods Section
**16**	Description of sample	Important characteristics of the sample? (e.g., demographics, date)	Methods Section
**Data Collection**			
**17**	Interview guide	Were questions, prompts, or guides used? Was it pilot tested?	Methods Section
**18**	Repeat interviews	Were repeat interviews carried out? How many?	Methods Section
**19**	Audio/visual recording	Was audio or video recording used?	Methods Section
**20**	Field notes	Were field notes made during/after the interviews?	Methods Section
**21**	Duration	What was the duration of the interviews/focus group?	File 5
**22**	Data saturation	Was data saturation discussed?	Methods Section
**23**	Transcripts returned	Were transcripts returned to participants for comment or correction?	Methods Section
**Domain 3: Analysis and Findings**			
**Data Analysis**			
**24**	Number of data coders	How many data coders coded the data?	Methods Section
**25**	Description of the coding tree	Did authors describe the coding tree?	File 6
**26**	Derivation of themes	Were themes pre-identified or derived from data?	Methods Section
**27**	Software	What software was used to manage the data?	Methods Section
**28**	Participant checking	Did participants provide feedback on findings?	Methods Section
**Reporting**			
**29**	Quotations presented	Were participant quotes presented and identified?	Results Section
**30**	Data and findings consistent	Was there consistency between data and findings?	File 6
**31**	Clarity of major themes	Were major themes clearly presented?	Table 1
**32**	Clarity of minor themes	Was there discussion of minor themes/diverse cases?	Discussion

File S5: Participant Demographics

Participant ID	Gender	Country based	Affiliated organisation type	Primary Expertise	Years of Experience	Interview Duration (hh:mm:ss)	Type of Stakeholder
**#1**	Male	England	Academia	Environmental Epidemiology	20	00:46:34	External
**#2**	Female	Wales	Public Health Institute	Population Health	28	00:30:48	Internal
**#3**	Male	Italy	International Public Health NGO	Environmental Prevention	41	01:12:15	External
**#4**	Male	Wales	Public Health Institute	Statistics and Public Health	38	00:41:03	External
**#5**	Male	Wales	Government	Environment	16	01:00:29	External
**#6**	Female	Switzerland	Intergovernmental Organisation	Tropical Medicine and Field Epidemiology	25	01:16:04	External
**#7**	Female	Scotland	Industry	Environmental Public Health	6	00:46:49	External
**#8**	Male	Wales	Public Health Institute	Emergency Planning, Preparedness and Response	9	00:29:36	Internal
**#9**	Female	England	Public Health Institute	Environmental Epidemiology	13	00:50:28	External
**#10**	Male	England	Public Health Institute	Public Health	36	00:52:30	External
**#11**	Female	Wales	Public Health Institute	Health Improvement and Public Health	17	00:35:37	Internal
**#12**	Female	Wales	Public Health Institute	Health Protection and Public Health	16	00:48:06	Internal
**#13**	Male	Canada	Intergovernmental Organisation	Veterinarian Epidemiology	38	01:46:38	External
**#14**	Male	Wales	Public Health Institute	Surveillance and Epidemiology	19	00:44:06	Internal

**#15**	Female	Belgium	Intergovernmental Organisation	One Health and Field Epidemiology	31	00:55:56	External
**#16**	Female	Scotland	Public Health Institute	Field Epidemiology	16	00:57:33	External
**#17**	Male	Switzerland	Intergovernmental Organisation	Field Epidemiology	32	00:55:41	External
**#18**	Female	France	Intergovernmental Organisation	Methodological Expert	38	01:13:45	External

Note. "Type of stakeholder" denotes the participant's relationship to Public Health Wales at the time of interview: Internal = PHW staff; External = non-PHW (other public health institutes, intergovernmental organisations, government, academia, industry, NGOs).

File S6: Word Cloud of the interview transcripts

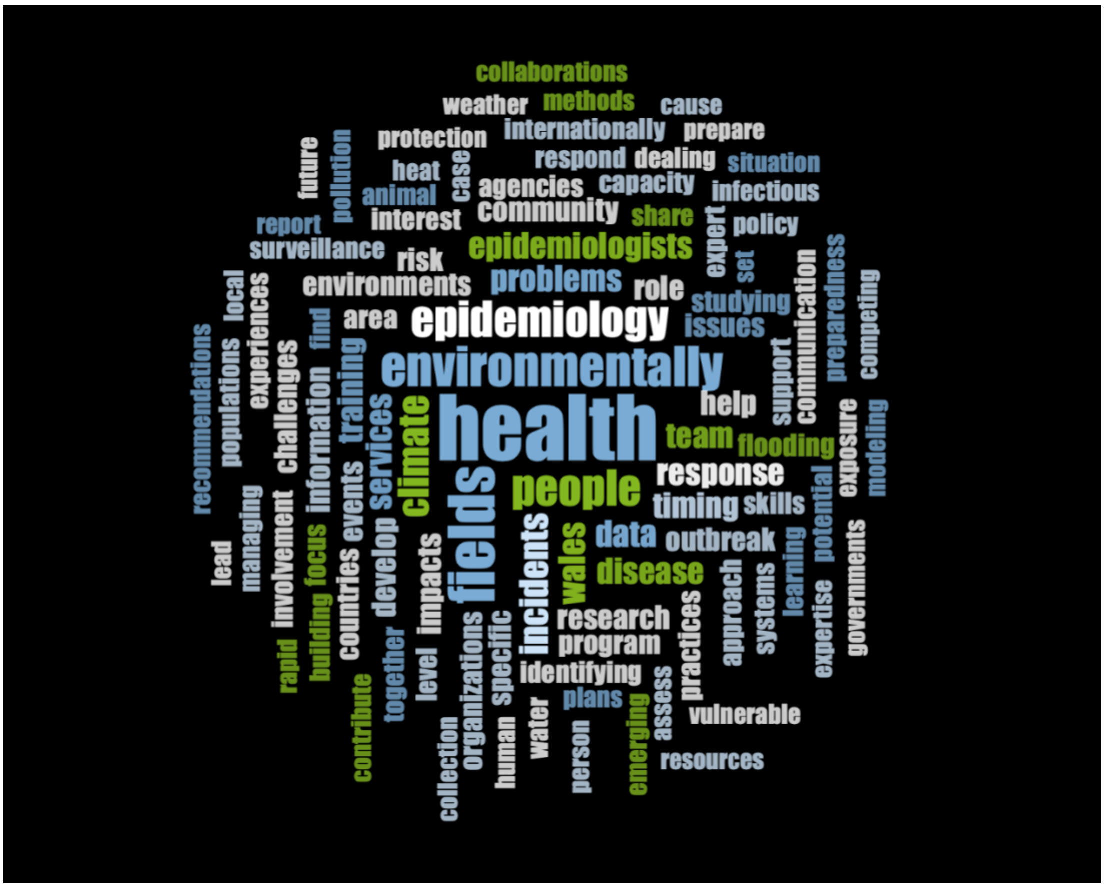

File S7: Detailed Codebook of the identified themes

Themes/Sub-themes	Description	Code Count
**Best Practices in Environmental & Climate Field Work**		68
**One Health Integration**	A cross-sectoral approach integrating human, animal, and environmental health was highlighted as a core principle of effective field epidemiology.	26
**Proof-of-Concept Pilots**	Small-scale interventions such as pilot heat or pollution monitoring projects were seen as useful starting points for expanding field epidemiology methods.	12
**Rapid, Evidence-Based Assessments**	Fast, data-driven evaluations using real-time health data (e.g., NHS 111, hospital admissions) were consistently described as critical for immediate action during events.	23
**Use of Established Tools**	Participants emphasized using frameworks like CASPER, environmental mapping, and pre-built surveillance templates, systems approach to ensure timely and structured responses.	7
**Common Challenges & Barriers**		123
**Attribution & Complexity**	Difficulty attributing specific health outcomes to climate/environmental causes due to multicausality and statistical uncertainty was commonly noted.	27
**Communication Barriers and Public Trust**	Challenges in public communication and building trust were commonly identified as barriers to effective fieldwork.	24
**Data Quality and Bias**	Many cited retrospective analysis, ethical delays, underreporting, and methodological shortcuts as limitations that hinder timely or accurate environmental health investigations.	22
**Resource Constraints and Structural Barriers**	Issues included under-resourced teams, lack of designated field epi units, and constraints on workforce or funding.	21
**Siloed Mandates & Expertise Gaps**	Fragmented systems and lack of environmental epidemiology expertise—particularly in public health training—were recurrent concerns.	29
**Equity, Vulnerable Populations & Risk Communication**		48
**Community Participation and Empowerment**	Participants highlighted the value of engaging affected communities directly in the field investigation process.	7
**Identify High-Risk Groups**	Interviewees consistently emphasized the importance of reaching groups like the elderly, displaced persons, or those not connected to health systems.	24
**Tailored Communication & Trust Building**	Cultural sensitivity, communication clarity, and building public trust were seen as crucial for effective fieldwork and public health action.	17
**Essential Skills & Competencies**		52
**One Health Competences**	Interdisciplinary collaboration, shared training and environmental training was emphasized.	14

**Soft Skills**	Communication, empathy, cultural competence, and adaptability were often named as essential, especially in crisis or community-facing roles.	21
**Technical and Operational Skills**	Descriptions included data management, GIS, environmental surveillance, survey design, and modelling, analysis.	17
**Expanding the Role of Field Epidemiology beyond Outbreaks**		69
**Application in Environmental and Climate Events**	Experts strongly supported applying field epidemiology methods to environmental hazards, citing floods, heatwaves, pollution, etc.	40
**Support During Recovery Phase**	Follow-up analysis, long-term health impact assessment, and system evaluation post-incident were commonly noted.	29
**Future Directions**		45
**Climate Modelling in Epidemiology**	Field epidemiology's role in health impact prediction and planning under different climate scenarios was acknowledged.	6
**Holistic Health Security**	Interviewees called for a systems-level approach that integrates surveillance, prevention, preparedness, and resilience across health and environmental domains.	31
**Technological & Methodological Innovation**	Calls for better exposure models, AI, environmental monitoring platforms, and integration with existing datasets were present.	8
**Intersectoral Collaboration is Critical**		64
**Global & Cross-Agency Partnerships**	Interviewees urged engagement with international networks and public health bodies in other countries for knowledge exchange and co-development.	37
**Local & National Coordination**	Multi-agency coordination at the national and sub-national level (e.g., PHW, local councils, emergency services) was cited as fundamental for response readiness.	27

File S8: Supplementary Quotations

Theme	Sub-Theme	Participant	Quotation
Best Practices in Environmental & Climate Field Work	One Health Integration	ID #3	*"Worked with many different disciplines... including biology, chemistry, physics, engineering, and even meteorology."*
Best Practices in Environmental & Climate Field Work	Rapid, Evidence-Based Assessments	ID #15	*"Quick recommendations without full evidence can lead to misjudgements… early pandemic messaging around ventilation is a lesson."*
Common Challenges & Barriers	Attribution & Complexity	ID #4	*"We need better, more precise measurement of environmental exposures to truly understand their health impacts."*
Essential Skills & Competencies	Soft Skills	ID #6	*"Listening is the most important skill. Keeping an open mind, not jumping to conclusions, applying scientific rigor."*
Essential Skills & Competencies	Technical and Operational Skills	ID #17	*"Field epi training needs to bring people back to the field…. not just R and statistics… real-world assessments."*
Expanding the Role of Field Epidemiology Beyond Outbreaks	Application in Environmental and Climate Events	ID #5	*"Field epi definitely applies… after floods, chemical incidents, or displacement, we need to assess population health."*
Expanding the Role of Field Epidemiology Beyond Outbreaks	Application in Environmental and Climate Events	ID #11	*"Climate change is the cause… but we're looking to understand the impacts and secondary causes… like mental health impacts post-flooding."*
Expanding the Role of Field Epidemiology Beyond Outbreaks	Application in Environmental and Climate Events	ID #2	*"A GP might notice that a certain community is reporting a higher rate of urinary tract infections... We would then look to field epi to say, can you have a look at the rate… has it flooded?"*
Expanding the Role of Field Epidemiology Beyond Outbreaks	Application in Environmental and Climate Events	ID #10	*"In Pakistan, after floods… field epi fellows worked on mapping vulnerabilities, designing welfare exemptions… not just infection control..."*
Expanding the Role of Field Epidemiology Beyond Outbreaks	Support During Recovery Phase	ID #10	*"We tried to measure mental health effects during the oil spill event, though we didn't go far enough."*
Future Directions	Holistic Health Security	ID #18	*"The future is bleak without multilateral cooperation, but linking diseases to lived environmental impacts may help mobilise change."*
Intersectoral Collaboration Is Critical	Global & Cross-Agency Partnerships	ID #6	*"Join international working groups to avoid reinventing solutions… share and access best practices in tracking, heatwave response, flooding, and surveillance."*
Intersectoral Collaboration Is Critical	Global & Cross-Agency Partnerships	ID #17	*"Good investigation requires hydrogeologists, atmospheric scientists, medical anthropologists… beyond health."*
Intersectoral Collaboration Is Critical	Global & Cross-Agency Partnerships	ID #8	*"We should learn from others. Less developed countries might have more experience managing catastrophic climate impacts."*
Intersectoral Collaboration Is Critical	Local & National Coordination	ID #7	*"We're working across modelling, fire and rescue, and air quality teams… all hold part of the picture."*
